# 14- to 16-Month-Olds Attend to Distinct Labels in an Inductive Reasoning Task

**DOI:** 10.3389/fpsyg.2017.00609

**Published:** 2017-04-24

**Authors:** Jessica L. Switzer, Susan A. Graham

**Affiliations:** Owerko Center and Department of Psychology, University of Calgary, CalgaryAB, Canada

**Keywords:** inductive inferences, categorization, infancy, inhibitory control, working memory, vocabulary

## Abstract

We examined how naming objects with unique labels influenced infants’ reasoning about the non-obvious properties of novel objects. Seventy 14- to 16-month-olds participated in an imitation-based inductive inference task during which they were presented with target objects possessing a non-obvious sound property, followed by test objects that varied in shape similarity in comparison to the target. Infants were assigned to one of two groups: a *No Label* group in which objects were introduced with a general attentional phrase (i.e., “Look at this one”) and a *Distinct Label* group in which target and test objects were labeled with two distinct count nouns (i.e., *fep* vs. *wug*). Infants in the Distinct Label group performed significantly fewer target actions on the high-similarity objects than infants in the No Label group but did not differ in performance of actions on the low-similarity object. Within the Distinct Label group, performance on the inductive inference task was related to age, but not to working memory, inhibitory control, or vocabulary. Within the No Label condition, performance on the inductive inference task was related to a measure of inhibitory control. Our findings suggest that between 14- and 16-months, infants begin to use labels to carve out distinct categories, even when objects are highly perceptually similar.

## Introduction

Naming plays a critical role in infants’ categorization and inductive reasoning, helping infants to unite diverse objects into categories and guiding their inferences about the shared properties of category members (e.g., Waxman and Booth, 2001; Welder and Graham, 2001; Graham et al., 2004; Plunkett et al., 2008; Ferry et al., 2010). Naming can also shape infants’ formation of distinct categories: that is, labeling objects with distinct labels facilitates object individuation and helps infants to divide objects into different categories (Waxman and Braun, 2005; Graham et al., 2013; Althaus and Westermann, 2016; Havy and Waxman, 2016). Here, we examined 14- to 16-month-old infants’ developing abilities to use labels to carve highly similar objects into distinct categories in an inductive inference task and the related abilities that may support this achievement.

Category-based inductive reasoning follows the premise that if something holds true for one exemplar of a category, we can reason that it will also hold true for other members of the same category. For example, one might observe that an entity has a particular property (e.g., a salmon swims), determine that two entities belong to the same category (e.g., salmon and tuna are both fish); infer that the second entity also exhibits that particular property (e.g., therefore, tuna also swim). Research has demonstrated that basic forms of inductive reasoning emerge early in development; infants between 9- and 11-months form category-based inductive inferences about the shared properties of animate (McDonough and Mandler, 1998; Vukatana et al., 2015) and inanimate objects (Baldwin et al., 1993).

The most commonly used methodology to assess infants’ inductive reasoning abilities relies on imitation paradigms (e.g., Baldwin et al., 1993). An experimenter models an action on a target object which elicits a non-obvious property. Infants are then presented with test objects that vary in similarity to the target object. If the infant infers that the test objects are members of the same category as the target, they should imitate the target action on the test object. Using this methodology, research has demonstrated 13- to 22-month-old infants will rely on shape similarity to guide their inferences about shared properties; that is, infants infer that objects that are highly similar in shape also share non-obvious properties (Welder and Graham, 2001; Graham et al., 2004; Graham and Diesendruck, 2010). If, however, target and test objects are introduced using the same count noun label, infants reduce their reliance on shape similarity and reason that even highly dissimilar objects share a non-obvious property (Welder and Graham, 2001; Graham et al., 2004; Graham and Kilbreath, 2007; Keates and Graham, 2008). This reliance on shared labels to guide inductive reasoning is selective: By 16-months of age, only novel words that are presented by a live speaker (vs. a recorded instruction), presented with a referential phrase (vs. presented alone), and clearly marked as count nouns (vs. adjectives) guide infants’ inferences that objects share properties (Keates and Graham, 2008). Taken together, this body of evidence indicates that when two perceptually *distinct* objects are labeled with the same count noun, infants as young as 13-months de-emphasize perceptual similarity and use the shared count noun label to guide their inferences.

In this study, we focus on another role of labels for young infants, namely, the use of labels to sort highly similar objects into distinct categories. Consider, for example, a situation in which an infant sees both a crow and a bat. Both animals are similar in size and shape, have wings, and fly—how do infants come to understand that bats and crows belong to two distinct categories? In cases such as these, one of the means by which accurate category membership may be gauged is through language input (e.g., Jaswal, 2007; Jaswal and Markman, 2007; Jaswal et al., 2009). That is, one way that people may learn that a bat does not belong to the bird category and is, in fact, a mammal, is by hearing different labels. Studies have demonstrated that naming objects with distinct labels supports the establishment of distinct categories in infants as young as 9 months of age (e.g., Waxman and Braun, 2005; Althaus and Westermann, 2016). In one recent study, Havy and Waxman (2016) presented 9-month-olds with novel animate-like creatures that varied along a perceptual continuum. When creatures were named with one count noun, infants formed a single category. In contrast, when one end of the continuum was labeled with one noun and the other end with a second noun, infants established two distinct categories.

In the context of inductive inference tasks, studies suggest a slightly different development emergence of sensitivity to distinct labels during the infancy years. When similarly shaped objects are labeled with two distinct count nouns, 13-month-olds appear to ignore the label information and continue to infer that same-shaped objects share properties (Graham et al., 2004). In contrast, 15-month-olds will de-emphasize perceptual similarity when objects are labeled with distinct labels. When infants were presented with similar shaped objects that were labeled with distinct count nouns (e.g., “This is not a *fep*. This is a *wug*.”), 15-month-olds de-emphasized the more salient cue of shared shape, and limited their generalization of the non-obvious property (Graham et al., 2013). Together, these findings suggest that there is a developmental change between 13 and 15 months in infants’ understanding and use of distinct labels in inductive reasoning tasks. What is less understood is which individual factors may contribute to this developmental shift. In this experiment, we tested 14- to 16-month-old infants and examined the potential influence of infants’ developing language abilities and executive function skills, such as working memory and inhibitory control, on their ability to de-emphasize perceptual similarity and use distinct labels to establish two distinct categories. Specifically, given the cognitive demands of privileging a distinct label over shape similarity, we asked whether infants’ increasing vocabulary size, age, and developing working memory and inhibitory control skills may contribute to infants’ ability to use distinct labels.

We had two specific goals in this experiment. First, we examined 14- to 16-month-olds’ use of distinct labels in an inductive reasoning task. By examining 14- to 16-month-old infants in the same study, we sought to better understand the developmental emergence of infants’ ability to favor distinct labels over shape similarity. Using a generalized imitation paradigm (see Keates and Graham, 2008), infants were presented with three sets of novel target objects that possessed non-obvious sound properties followed by a test object that varied in shape similarity relative to the target objects (i.e., high- and low-similarity). Each infant was presented with one of the three object sets in one of three within-subject expectation conditions: the *violated* condition, the *baseline* condition, and the *predicted* condition. The condition of interest was the *violated* condition, in which the target object had the non-obvious sound property, but the test object was disabled. If infants expect the test object belongs to the same category as the target object, they will attempt to elicit the non-obvious sound property on the disabled test object. The baseline condition, in which the target and test object’s non-obvious property was disabled, provided a measure infants’ exploratory actions on the objects. In the *predicted* condition, both the target and test object contained the non-obvious property. This condition was included to ensure that infants did not become frustrated by continually attempting to elicit the sound property without success (as is the case in the violated condition). Infants were tested in one of two label groups (i.e., *No Label, Distinct Label*). In the *No Label* group, the experimenter introduced the target and test objects using a general phrase (e.g., “Look at this one!”). In the *Distinct Label* group, the experimenter introduced the target and test objects using distinct count nouns [e.g., “This is a *wug*.” (target) and “This is a *blick*. This is not a *wug*” (test)].

The second goal was to examine individual factors which may be contributing to infants’ developing abilities to use distinct labels. As suggested by previous research, age is likely a factor contributing to infants’ ability to use distinct labels to establish distinct categories and reason about their shared properties (Graham et al., 2004, 2013). What has not yet been investigated is whether other abilities may be contributing to this developmental shift. Here, we specifically examined the role of individual differences in executive function and vocabulary size in infants’ ability to privilege distinct labels in the presence of highly similar objects. In terms of executive function, we examined the roles of inhibitory control and working memory. Inhibitory control is the ability to suppress automatic approach behavior when it is situationally inappropriate or when explicitly directed to not engage in an automatic behavior (Diamond and Gilbert, 1989; Diamond and Taylor, 1996). The ability to inhibit competing responses and resist interference is essential, as it reduces the cognitive load and allows for quicker and more efficient processing of information (Garon et al., 2008). Inhibitory control is one of the most extensively studied executive functions in the preschool years, yet research regarding inhibitory control in infancy is still emerging. When considering the use of distinct labels in inductive reasoning, infants must prioritize the linguistic information over perceptual information and inhibit their dominant response to generalize non-obvious properties to objects that are highly perceptually similar. To assess inhibitory control, we used a modified version of the detour-reaching task (Yott and Poulin-Dubois, 2012). The detour-reaching task has been established as a reliable method to assess infants’ complex inhibition skills.

Working memory refers to the system of memory that allows individuals to simultaneously process and store information (Baddeley and Hitch, 1974; Baddeley, 1986). Working memory is argued to be the first component of executive functions to begin developing, as the ability to hold information in the mind over a delay is fundamental in order to carry out more sophisticated executive functions (Garon et al., 2008). Working memory begins to develop prior to 6 months of age (Pelphrey and Reznick, 2003), continues to develop throughout childhood, and peaks in performance approximately at the age of 20 (Letho et al., 2003; Huizinga et al., 2006). Given the memory demands of holding distinct labels in mind, we examined whether working memory may play a crucial role in infants’ ability to use different labels. We used the hide-the-pots task as a measure of working memory in the current study (Bernier et al., 2010).

Finally, we examined the potential contribution of infants’ developing vocabulary to the use of distinct labels. There is significant growth in number of words in vocabulary between 14 and 16 months of age, as the average productive vocabulary size more than doubles over these 2 months (Fenson et al., 2006). Furthermore, some research suggests that as infants become more reliant on labels to form categories, their vocabulary improves in a similar pattern (e.g., Nazzi and Gopnik, 2000). Thus, it is suggested that as children learn more words, they begin to understand that distinct labels denote specific categories.

We have two sets of predictions. The first set focus on infants’ performance on the induction task, and vary according to the similarity of the test objects. That is, we predicted that infants in the Distinct Label group would inhibit their generalization of non-obvious properties to the high-similarity object, and thus, perform significantly fewer target actions on the high-similarity object when compared to the No Label group, following from literature suggesting that distinct labels designate members of distinct categories (e.g., Graham et al., 2013; Havy and Waxman, 2016). Our predictions for the low-similarity objects are more exploratory: it is possible that hearing the distinct label would lead infants to inhibit their generalization to the low-similarity object relative to the No Label group. Given, however, that there is little information provided by this object to suggest shared category membership for infants in the No Label group, it is possible that there will be no differences in performance of action across both groups.

The next set of predictions focus on the potential contribution of individual differences with respect to infants’ use of distinct labels in the inductive reasoning task. First, we expected that infants’ use of distinct labels would be related to age, based on previous research (e.g., Graham et al., 2004, 2013). Specifically, we expected that the tendency to perform actions on the high-similarity object would decrease with age across the 14–16 month age range tested in the study. Furthermore, we expected that infants who performed better on the inhibitory control and working memory measures, and infants with a greater productive vocabulary would rely more on the distinct label compared to infants who performed more poorly on these individual differences meaures.

## Materials and Methods

### Participants

The final sample was comprised of 70 14- to 16-month-old infants assigned to one of two conditions: the Distinct Label condition (*n* = 35) or the No Label condition (*n* = 35). See **Table 1** for mean age, gender, vocabulary size, and parent education. An additional 25 infants were tested, but excluded from analysis for the following reasons: excessive fussiness leading to failure to complete all the tasks (*n* = 10), parental interference (*n* = 2), parent-reported developmental atypicalities (*n* = 2), technical difficulties (*n* = 2), and statistical outliers (see description below; *n* = 9). All infants were born full term and were from homes in which English was the primary language spoken. This study was approved by the Conjoint Faculties Research Ethics Board at the University of Calgary. Parental consent for participation was obtained in writing prior to the testing session.

**Table 1:** Infant age, vocabulary, gender, and parental education as a function of condition.

	No Label	Distinct Label
Age**^∗^**		
Mean	15.64 (0.80)	15.48 (0.85)
Range	14.16 – 16.89	14.10 – 16.95
Gender	15 Male	18 Male
	20 Female	17 Female
CDI^∗∗^		
Mean	26.36 (25.17)	19.21 (19.71)
Range	0.00 – 98.00	0.00 – 79.00
Hide the Pots Task		
Mean	1.14 (0.69)	1.23 (0.69)
Range	0.00 – 3.00	0.00 – 3.00
Detour-Reaching Task		
Mean	5.20 (2.36)	5.88 (1.60)
Range	0.00 – 8.00	3.00 – 8.00
Parental Education^∗∗∗^ (%)		
Elementary	1.5	0.0
High School	14.2	10
College/Undergraduate	74.3	62.9
Graduate Degree	7.1	22.8
Other	1.4	2.9

*^∗^ Age, age in months. ^∗∗^ CDI, number of words understood and produced based on parental report on the MacArthur-Bates CDI. ^∗∗∗^ Parental Education, maternal and paternal education. Infants in the Distinct Label and No Label group did not differ significantly in age, CDI score, performance on the Hide the Pots task, performance on the Detour-reaching task, or parental education, *t*(68) = 0.78, *p* = 0.437, *t*(52) = 3.13, *p* = 0.083, *t*(68) = 0.52, *p* = 0.61, *t*(68) = -1.42, *p* = 0.16, and χ^*2*^(3) = 0.19, respectively.*

### General Procedure

Infants were presented with three tasks during the testing session: an inductive inference task, an inhibitory control task (Detour-Reaching Task), and a working memory task (Hide the Pots Task). The order of the tasks was counterbalanced across infants to address any potential carryover effects. During the three tasks, the infant sat either in a high chair or on their parent’s lap. Parents were instructed not to interfere with any of the tasks. Parents were also instructed to replace the objects on the table, directly in front of the infant, in the event that the infant dropped an object off the table or passed the object to them. For the purposes of coding, sessions were videotaped with a Sony HDR-CX240 HD Handycam Camcorder. Trials were timed with a handheld stopwatch.

### Inductive Inference Task

#### Materials

**FIGURE 1:**
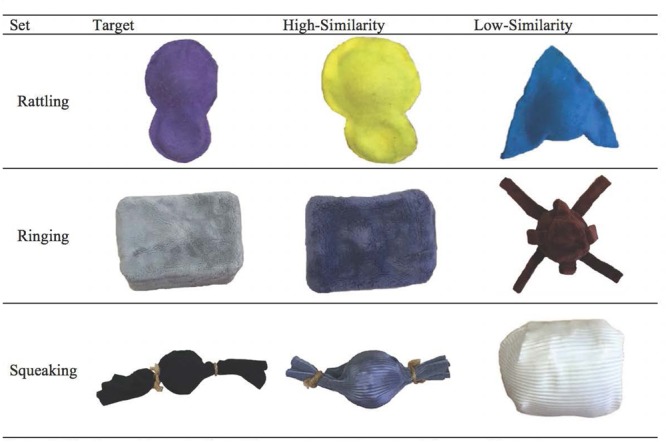
**The three object sets: the ringing set, the rattling set, and the squeaking set.** Within each object set there was a target object (top center), high-similarity object (bottom left), and low-similarity object (bottom right).

There were three objects presented in the warm-up phase: a clothesline pulley, a turning clock, and a plastic garlic press. Stimuli in the test phase consisted of three object sets: a ringing set, a rattling set, and a squeaking set (see **Figure 1**). Each set included a target object, a high-similarity test object, and a low-similarity test object. The high-similarity objects were the same shape and texture as the target object, but differed in size and color. The low-similarity objects were different in shape, size, and color, but shared the same texture as the target object. Two versions of each of the object sets were created: a functional version in which the target and test objects possessed a non-obvious sound property that could be elicited by performing a specific target action and a disabled version in which the objects did not have the non-obvious property (i.e., did not make a sound).

The ringing set objects consisted of a metal bell (7 cm in diameter) placed inside of a Styrofoam rectangular shaped box covered with a soft, plush material. When tapped, the functional version rang, whereas the disabled version remained silent. The rattling set objects were rattlers (7 cm rattle × 4 cm handle) covered with felt. When shaken, the functional version rattled, whereas the disabled version was silent. The squeaking set objects were hollow rubber balls (7 cm in diameter) covered with pleated rayon and tied together with a string. When squeaked, the functional version squeaked, whereas the disabled version remained silent.

#### Design

Infants were randomly assigned to one of two label groups: (a) *No Label group*, or a (b) *Distinct Label group* (see procedure below). For each infant, one of the three object sets was presented in one of three within-subjects expectation conditions: the baseline condition, the violated condition, and the predicted condition (see **Table 2**). The condition of primary interest was the violated-expectation condition, in which the target object elicited a non-obvious property but the test object did not. This condition was used to assess whether infants expected the target and test objects to share the same non-obvious property. If infants infer that the test object and target objects belong to the same category, they should persist in performing the target action on the target object to elicit the non-obvious property. The baseline condition, in which both the target and the test objects were disabled, was used to assess infants’ exploratory actions with the objects. The predicted condition, in which both the target and test objects were functional, was used to maintain infants’ interest in the objects so that they did not become frustrated. In keeping with previous research (e.g., Baldwin et al., 1993; Welder and Graham, 2001; Graham and Kilbreath, 2007), the data from the predicted condition will not be analyzed, as it is difficult to interpret whether infants had an expectation about the property, or whether they continue to elicit the property due to the reinforcing nature of performing the target action. For each test trial the target object and either the high- or low-similarity object were presented.

**Table 2:** Summary of the three within-subject conditions in the inductive inference task.

Condition	Infants’ expectation	Presence of the non-obvious property	
		Target object	Test object
Baseline	None	Absent	Absent
Violated	Violated	Present	Absent
Predicted	Fulfilled	Present	Present

*Infants’ expectation column summarizes infants’ expectation about the non-obvious property in the test object for each given condition.*

Test trials were presented in two blocks with each block consisting of three test trials (one from each of the violated, baseline, predicted conditions). An object from each set was presented in each block (see **Table 3** for example testing protocol). The order of the presentation of the test objects within each block and the order of the presentation of conditions were counterbalanced across participants. Each testing protocol was yoked across groups.

**Table 3:** Example testing protocol.

Block	Trial	Condition	Object set	Test object similarity
1	1	Violated	Ringing	Low-similarity
	2	Baseline	Rattling	Low-similarity
	3	Predicted	Squeaking	High-similarity
2	4	Violated	Ringing	High-similarity
	5	Baseline	Rattling	High-similarity
	6	Predicted	Squeaking	Low-similarity

#### Procedure

The task began with three warm-up trials designed to demonstrate to infants that they should imitate the experimenter’s actions. The experimenter first demonstrated a target action on warm-up objects, and then asked the parent to do the same. After demonstrating the action, the parent passed the object to their infant. All infants imitated at least one of the three actions with the object.

**FIGURE 2:**
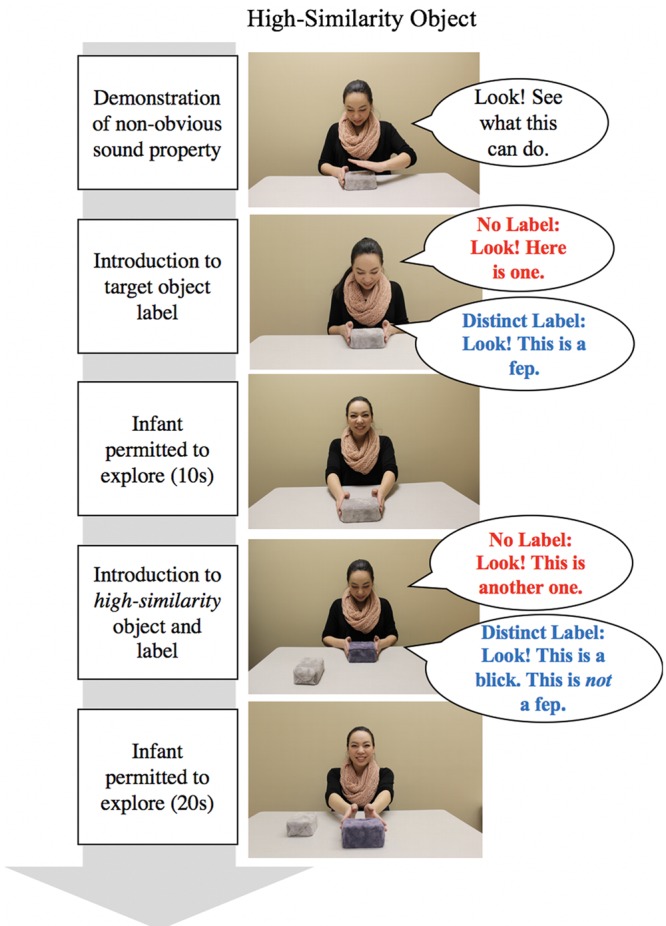
**Overview of inductive inference test procedure for high-similarity test objects with example dialog.** Please see “Procedure” section for full details of the dialog.

**FIGURE 3:**
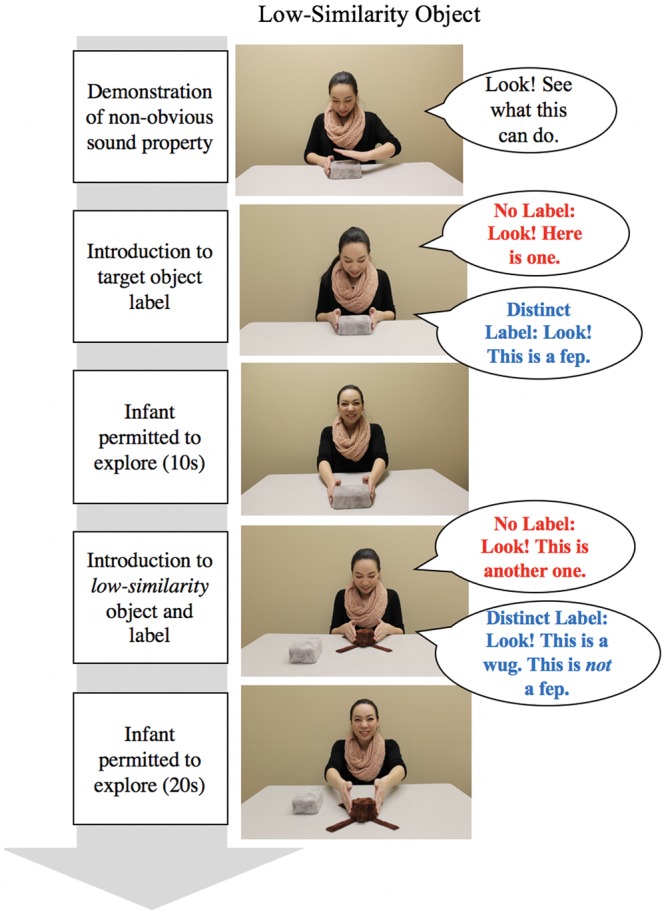
**Overview of inductive inference test procedure for low similarity test objects with example dialog.** Please see “Procedure” section for full details of the dialog.

See **Figures 2, 3** for an overview of the procedure for the test trials. At the start of each test trial, the experimenter placed the target object in front of the infant, but out of reach, and drew the infant’s attention to the object (e.g., *“Look. Look at this.”*). For test trials in the predicted and violated conditions, she then demonstrated how the target action produced the non-obvious property (e.g., hit the top of the ringing object to evoke the sound while saying *“Look. See what this can do.”*). The action was demonstrated five times in a row. For test trials in the baseline condition, no actions were demonstrated.

Following this initial introduction to the target objects, the procedure then diverged according to label group. In the No Label group, the experimenter drew attention to the target object with a general attentional phrase (e.g., *“Look at this one.”*). In the Distinct Label group, the experimenter labeled the target object using a count noun label (e.g., *“Look! This is a fep.”*).

Following the attentional phrase or labeling phrase, the infant was permitted to explore the object for 10 s. The target object was then placed on the table within the infants’ view, but out of reach. Next, the experimenter introduced the test object with either a general attention phrase (No Label group: *“Look! This is another one.”*) or with a distinct novel count noun than was used for the target object (Distinct Label group: “Look! This is a *wug*. Here is a *wug*… This is not a *fep*.”). For each introduction to the target and test object, the novel count noun or attentional phrase was repeated six times. The language prompts used in the No Label and Distinct Label groups were chosen as they were similar to those used in previous inductive reasoning studies (e.g., Keates and Graham, 2008; Graham et al., 2013). Following the introduction of the test objects, the infant was permitted to explore the object for 20 s. This test procedure was repeated six times using the high- and low-similarity objects in each of the three conditions (baseline, violated, predicted).

#### Coding and Data Screening

The number of target actions infants performed on the target and test objects were recorded by trained coders who were unaware of the experimental hypothesis and group assignment. Coders were not be able to distinguish the expectation conditions from one another as videos were coded with the sound turned off. Target actions for the ringing set consisted of a rapid tapping or patting motion performed with the hand (i.e., infant brought his or her hand down to make contact with the object). If the infant tapped the object with two hands simultaneously, this was coded as a single action. Exploring or poking the top of the object in the absence of a tapping motion was not considered a target action. The target action for the rattling set consisted of a shaking motion (i.e., back and forth, upward or downward motion) with the object in the infants’ hand. If the infant performed a continuous back and forth or up and down motion, this was considered a single target action, however, if there was a delay between the two motions (i.e., shaking the object one direction, then in another direction), this was considered two target actions. Infants could shake the object with one or two hands. Manipulating the object in order to throw/pass it to the parent or examiner did not constitute a target action. The target action for the squeaking set consisted of a squeezing motion on the object with one or both hands. To constitute a target action, the infants’ fingers had to contract around the object. Releasing the object was not considered a second action. Coders also recorded the frequency and type of object transfer actions, which were defined as performing a target action from one object set (e.g., the squeezing action from the squeaking set) on a test object of a different set (e.g., the ringing set).

Twenty-one percent of the data (*n* = 15 randomly selected participants) was re-coded by another research assistant, unaware of the experimental hypothesis. Interclass coefficients (ICCs) for frequency of target actions on the target objects and test objects were all above 0.98 (all *ps* < 0.001).

Infants whose standard scores for frequency of the target action were greater than 3.0 standard deviations above or below the mean in the violated and baseline condition were considered outliers and thereby removed from the data analysis (No Label Group: *n* = 5; Distinct Label Group: *n =* 4). Multivariate outliers were examined by calculating the Mahalanobis distance and comparing to a critical value of 0.05 (df = 3). There were no significant multivariate outliers (*n* = 0).

### Detour-Reaching Task

The detour-reaching task was used to assess infants’ inhibition abilities. This task involved infants seeing a desirable object inside of a wooden box through a transparent window. In order to successfully retrieve the object, infants had to turn a knob on the side of the box that opened the window, as demonstrated by the experimenter. Accordingly, there were two phases during this task: a demonstration phase and a testing phase. A direct reach (the prepotent response) toward the object through the transparent window resulted in a failure on the task. The detour-reaching task used in the current study has been established as a reliable measure of complex inhibition in infants 18- to 24-months of age (e.g., McGuigan and Nunez, 2006; Esseily et al., 2010; Garon et al., 2014). The specific procedure and design used in this study was adapted from the detour-reaching task used by Yott and Poulin-Dubois (2012).

#### Materials

**FIGURE 4:**
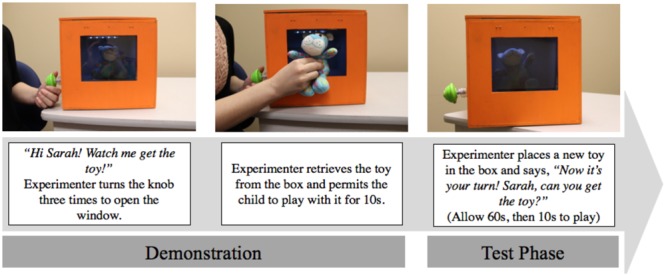
**Detour-reaching task: demonstration and test phase**.

The materials for this task included an orange wooden box (30 cm width, 26.5 cm height, 37 cm depth), a hand-held remote, and four small stuffed toys. On the front of the box, there was a centered, rectangular cut out (16 cm width, 13 cm height; see **Figure 4**). A transparent, plexiglass window covered the opening. This window allowed the infants to see the inside of the box, but they were unable to directly retrieve the stuffed toy from inside the box. The window was attached to the inside of the box and could only be controlled by a remote control. There were two cylindrical metal locks, controlled by remote control that secured the window in the closed position to prevent infants from pushing through the plexiglass. A green knob was placed on the left-hand side of the box. There was a small push light located on the inside roof of the box that illuminated the inside of the box.

#### Procedure

See **Figure 4** for an overview of the procedure. There were two phases in this task: a demonstration phase and the test phase. During the demonstration phrase, the experimenter introduced the task by saying *“Hi (child’s name). Watch me get the toy.”*, as she turned the knob located on the left-hand side of the box three times. This appeared to open the window, which allowed the experimenter to retrieve the toy from the box. The experimenter was actually controlling the window via a remote control, not visible to the infant. The experimenter then gave the infant the toy to play with for 10 s. Following the demonstration, the experimenter turned the knob and opened the window to place the new toy inside the box. The experimenter then closed and locked the window (via remote control) while simultaneously turning the knob. To begin the test phase, the experimenter then said, *“Now its your turn. (Child’s name), can you get the toy?.”* The infant was given 45 s to retrieve the toy. Regardless of success, the child was then permitted to play with the toy for 10 s. If the infant did not proceed to touch the knob during the test phase, the experimenter removed the box from the infant’s view, removed the toy, and proceeded with the next test trial. The experimenter demonstrated putting the new toy inside the box and closing the window on each trial. This test trial was repeated four times with four different objects, and infants were awarded one point for touching the knob before touching the window on each trial. A prompt was provided after 10 s if the infant made no attempt to get the toy.

#### Coding

Coders, blind to the hypothesis, scored infants as a two, one or zero based on whether they touched the knob or window first. Infants were awarded two points if they touched the knob before the window, one point if they touched the window before the knob or zero points if they never touched the knob throughout the duration of the 45 s trial. As there were four test trials, infants could receive a total score between zero and eight. ICCs for 21% of the data (*n* = 15 randomly selected participants) were all above 0.99 (*ps* < 0.001).

### Hide the Pots Task

The Hide the Pots task was administered to obtain a measure of infants’ working memory abilities. This task involves hiding a toy under one of three pots, covering the pot with a blanket, and asking the infant to retrieve the toy after a 5s delay.

#### Materials

**FIGURE 5:**
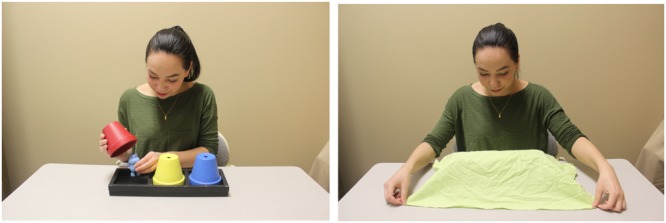
**Hide the Pots task: experimenter hiding the toy under one of the opaque pots and covering display with the blanket**.

The materials for this task included three painted clay pots (red, yellow, blue) set on a wooden potholder, a small, green baby blanket, and four small plastic toys (see **Figure 5**).

#### Procedure

This procedure was based on the task adapted by Bernier et al. (2010). See **Figure 5** for an overview of the procedure. A small plastic toy was hidden in full sight of the infant under one of three opaque pots, each differing in color. The warm-up phase involved asking the infant to immediately retrieve the toy from where it was hidden. This phase was intended to introduce the infant to what was to be expected in the test phase. In the test phase, the experimenter hid the toy under one of the pots, the pots were then covered with a blanket, and the infant was asked to find the toy. Thus, the infant had to hold the location of the sticker in their memory, remove the blanket, and then select the correct pot. This test trial was repeated three times, with the experimenter hiding the toy under each of the colored pots once for a total of three trials. The order in which the toy was hidden under the pots was counter-balanced across trials.

#### Coding

Coders, blind to the hypothesis of the experiment, scored infants as one or zero based on whether or not they grabbed the correct pot on the first try. A grab was defined as an intentional lift with one or both hands. A score of one indicated that the infant grabbed the correct pot on the first try. A score of zero indicated that the infant grabbed one of the incorrect pots on the first try. There were three test trials and thus infants could receive a total score of zero to three. The ICC for 21% of the data (*n* = 15 randomly selected participants) was 1.00 (*p* < 0.001).

### MacArthur-Bates Communicative Development Inventory (CDI): Words and Gestures

At the end of the study, parents were asked to complete the CDI to obtain a measure of infants’ vocabulary size. If parents opted to complete the questionnaire at home, they were asked to fill it out within 48 h, include the date it was completed, and return it using the provided stamped and addressed envelope. Parents received a reminder e-mail 1 week following their appointment if the CDI had not been received. 54/70 (77%) of CDIs were completed and returned.

## Results

In our first set of analyses, we examined infants’ performance on the inductive inference task, focusing on the baseline and violated-expectation conditions. Note that we did not analyze the data from the predicted condition as performance in this condition is difficult to interpret. That is, it is impossible to determine whether infants continue to perform the target action due to an expectation about the non-obvious property, or whether infants continue trying to elicit the non-obvious property due to the reinforcing nature of the sound property on the test objects (for further discussion of this issue see Baldwin et al., 1993; Welder and Graham, 2001). In the second set of analyses, we examined the relations between age, executive function and vocabulary size and infants’ performance on the inductive inference task.

### Inductive Inference Task Analyses

Preliminary analysis revealed that all infants in both the No Label and Distinct Label groups performed at least one target action during the warm-up phase. Similarly, 32/35 infants in the No Label group and 30/35 infants in the Distinct Label group performed at least one target action on the target object in both the violated and predicted conditions. This suggests that infants understood the instructions to imitate the experimenter’s target actions on the objects.

We next analyzed infants’ performance of actions during the baseline condition. Recall that this condition was included to provide a baseline measure of infants’ actions on the objects, in the absence of any demonstration by the experimenter. Inspection of the data indicated that the majority of infants in each group did not perform any target actions on the high- and low-similarity objects in the baseline condition and thus, the data were analyzed using non-parametric methods. Specifically, 83% of infants in the No Label group and 86% of infants in the Distinct Label group had frequency of target action scores of 0 for the high-similarity test object in the baseline condition. Similarly, 80% of infants in the No Label group and 94% of infants in the Distinct Label group had frequency of target action scores of 0 on the low-similarity test object in the baseline condition. Performance of target actions in the baseline condition on the high- and low-similarity objects did not significantly differ across groups, χ^2^(3) = 1.42, *p* = 0.70, and χ^2^(3) = 5.41, *p* = 0.37, respectively.

**FIGURE 6:**
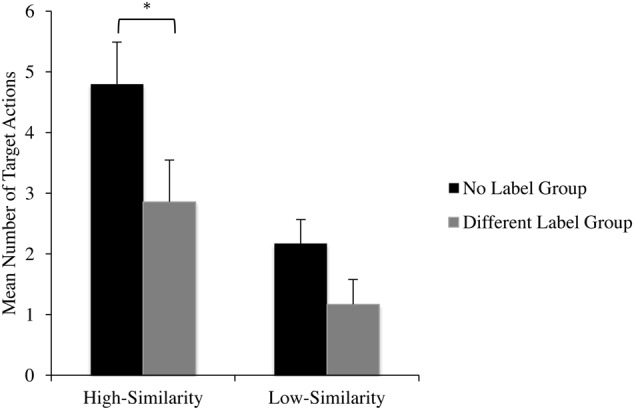
**Mean number of target actions performed on test objects as a function of similarity and label group.** ^∗^*p* < 0.05.

The primary analyses focused on infants’ frequency of target actions on the test objects in the violated condition. See **Figure 6** and **Table 4** for the mean number of target actions performed as a function of similarity and label group. To examine whether infants’ performance of target actions on test objects varied as a function of label group and shape similarity to the target object, we used a 2 (Label Group: no label, distinct label) × 2 (Shape Similarity: high, low) mixed factor ANOVA. The analysis revealed a main effect of label group, such that infants in the Distinct Label group (*M* = 2.01, *SD* = 2.71) performed significantly fewer actions on test objects than infants in the No Label group (*M* = 3.49, *SD* = 3.67), *F*(1,68) = 5.48, $\eta_{p}^{2}$ = 0.08, *p* = 0.02. There was also a significant main effect of similarity, with infants performing significantly more actions on the high-similarity object (*M* = 3.83, *SD* = 4.15) than the low-similarity object (*M* = 1.67, *SD* = 2.45), *F*(1,68) = 19.20, $\eta_{p}^{2}$ = 0.22, *p* < 0.001. The Label Group × Shape Similarity interaction was not significant, *F*(1,68) = 0.92, *p* = 0.34, $\eta_{p}^{2}$ = 0.01.

**Table 4:** Frequency of target actions performed on the test objects at each level of shape similarity within each expectation condition.

			Shape similarity to target			
	Target		High		Low	
Group	*M*	*SD*	*M*	*SD*	*M*	*SD*
No Label						
Violated	4.54	4.57	4.80	4.84	2.17	2.50
Baseline	0.31	0.29	0.29	0.71	0.57	1.33
Predicted	4.40	4.27	7.69	7.65	4.17	5.75
Distinct Label						
Violated	3.97	5.14	2.86	3.10	1.17	2.32
Baseline	0.34	1.08	0.23	0.60	0.06	0.24
Predicted	4.40	5.40	6.54	8.60	3.09	7.31

In considering the results from the ANOVA and specifically the lack of a significant interaction between label group and similarity, we note that a non-significant *F*-test can mask significant pairwise comparisons, a phenomenon that has been labeled non-consonance (Gabriel, 1969; Keppel and Zedeck, 1989; Hancock and Klockars, 1996). Thus, in the next set of analyses, we carried out planned comparisons to address our prediction that infants in the Distinct Label group would inhibit their generalization of non-obvious properties to the high-similarity object, and thus, perform significantly fewer target actions on the high-similarity object when compared to the No Label group. This analysis indicated that infants in the Distinct Label group (*M* = 2.86, *SD* = 3.10) performed significantly fewer target actions on the high-similarity objects than infants in the No Label group (*M* = 4.80, *SD* = 4.84), *t*(68) = 2.00, *p* = 0.049, *d* = 0.48. This is considered a moderate effect according to Cohen’s (1988) standards. Conversely, infants in the Distinct Label group (*M* = 1.17, *SD* = 2.32) and the No Label group (*M* = 2.17, *SD* = 2.50), performed a similar number of target actions on the low-similarity object *t*(68) = 1.46, *p* = 0.09, *d* = 0.4. These results suggest that when an object is labeled with a distinct label, infants are less likely to generalize target properties to highly similar test objects compared to when the object is not labeled with a count noun.

### Relations with Age, Executive Functions and Vocabulary

Next, we examined the relation between performance on the inductive inference task, age, vocabulary size, inhibitory control, and working memory. **Table 5** shows the correlations between age, vocabulary size, inhibitory control, and working memory for the overall sample. **Tables 6, 7** show the correlations in the No Label and Distinct Label groups, respectively. As one might expect, age was significantly positively correlated with working memory (*r* = 0.27, *p* = 0.025) and vocabulary size (*r* = 0.34, *p* = 0.01). Inhibitory control was significantly, positively correlated with infants performance on the high-similarity object in the No Label group. In the Distinct Label group, age was significantly, negatively correlated with infants’ performance on the induction task.

**Table 5:** Zero-order correlations between age, vocabulary size, working memory and inhibitory control for the overall sample

	Age	CDI	Memory	IC
Age	–	0.34^∗^ *n* = 53	0.27^∗^ *n* = 70	0.15 *n* = 70
CDI		–	0.13 *n* = 53	–0.04 *n* = 53
Hide the Pots (Memory)			–	0.00 *n* = 70
Detour-Reaching Task (IC)				–

*^∗^*p* < 0.05 (two-tailed). CDI, number of words understood and produced based on parental report on the MacArthur-Bates CDI; statistical outlier removed from CDI correlations (*n* = 1).*

**Table 6:** Zero-order correlations between age, performance on the inductive inference task, working memory and inhibitory control in the No Label Group only.

	Age	High	Low	CDI	Memory	IC
Age	–	0.22 *n* = 35	0.12 *n* = 35	–	–	–
Violated High Test (High)		–	0.35^∗^ *n* = 35	–0.23 *n* = 25	–0.14 *N* = 35	0.34^∗^ *n* = 35
Violated Low Test (Low)			–	0.00 *n* = 25	–0.03 *n* = 35	0.13 *n* = 35
CDI				–	–	–
Hide the Pots (Memory)					–	–
Detour-Reaching Task (IC)						–

*^∗^*p* < 0.05 (two-tailed). CDI, number of words understood and produced based on parental report on the MacArthur-Bates CDI.*

**Table 7:** Zero-order correlations between age, performance on the inductive inference task, working memory and inhibitory control in the Distinct Label Group only.

	Age	High	Low	CDI	Memory	IC
Age	–	–0.39^∗^ *n* = 35	–0.10 *n* = 35	–	–	–
Violated High Test (High)		–	0.16 *n* = 35	–0.08 *n* = 28	–0.07 *n* = 35	0.21 *n* = 35
Violated Low Test (Low)			–	0.37 *n* = 28	–0.19 *n* = 35	0.21 *n* = 35
CDI				–	–	–
Hide the Pots (Memory)					–	–
Detour-Reaching Task (IC)						–

*^∗^*p* < 0.05 (two-tailed). CDI, number of words understood and produced based on parental report on the MacArthur-Bates CDI; statistical outlier removed from CDI correlations (*n* = 1).*

Two hierarchical linear regression analyses, one for each label group, were conducted to identify the relative influence of the predictor variables (i.e., age, working memory, and inhibitory control) on the number of target actions infants performed on the high-similarity objects (there were no significant correlations with performance of actions on the low-similarity objects and thus no further analyses were conducted). Specifically, age was entered at Step 1 and the executive function measures (working memory and inhibitory control) were entered at Step 2. Infants’ vocabulary scores were not included in the analyses as we did not have complete data for the entire group. However, the correlations between infants’ vocabulary size and the number of target actions performed on the high-similarity objects were not significant in the No Label group or the Distinct Label group (see **Tables 6, 7**, respectively).

In the No Label group, the hierarchical linear regression revealed that in Step 1, age did not significantly contribute to the regression model, *R^2^* = 0.05, *F*(1,34) = 1.67, *p* = 0.21, nor did the addition of executive function measures in Step 2, *R^2^* = 0.15, *F*(3,34) = 1.78, *p* = 0.17. In the Distinct Label group, the analysis revealed a significant regression model in Step 1 with age as a predictor and in Step 2 with age, working memory, and inhibitory control as predictors (see **Table 8** for the regression statistics for the Distinct Label group). However, examination of the beta values demonstrate that age was the only statistically significant predictor suggesting that as age increases, infants perform significantly fewer target actions on the high-similarity target object. Working memory and inhibitory control did not significantly add to the regression model. In fact, a comparison of the Step 1 and Step 2 models for the Distinct Label group revealed no significant change. Taken together, this suggests that age, but not working memory or inhibitory control, was the only significant variable predictor of infants’ performance of target actions on the high-similarity objects.

**Table 8:** Summary of Hierarchical Regression Analysis for variables predicting the number of target actions performed on the high-similarity object in the Distinct Label group.

Variable	β	*b*	*SE*	*t*	*R*	*R*^2^	Δ*R*^2^
Step 1					0.39ˆ*	0.15ˆ*	0.15ˆ*
Age	0.39	–1.43	0.59	–2.43ˆ*			
Step 2					0.47ˆ*	0.22ˆ*	0.07
Age	–0.45	1.01	1.04	–2.55ˆ*			
Working Memory	0.09	–0.82	1.18	0.49			
Inhibitory Control	0.24	0.58	0.35	1.48			

*N = 35; ^∗^*p* < 0.05 (two-tailed).*

## Discussion

Our findings demonstrate that the ability to use distinct labels to form distinct categories emerges between 14- and 16-months of age. Furthermore, our findings indicate that age, perhaps as an index of general cognitive maturation, but not working memory, inhibitory control, or vocabulary, contributes to this developing ability. We discuss each of these findings in turn.

First, our results corroborate and extend the existing literature regarding infants’ inductive inferences and the use of labels. When test and target objects were labeled with distinct count nouns (e.g., *“This is a blick*… *This is not a wug.”*), 14- to 16-month-olds were significantly less likely to generalize the non-obvious property to the high-similarity test object than when objects were not labeled. Thus, when target and test objects were labeled with distinct labels, infants de-emphasized the importance of shape similarity amongst the two objects, suggesting that they appreciated that the objects belonged to distinct categories. This point is particularly compelling when one considers that the high-similarity test object was identical to the target object in all aspects except color. These results are consistent with a large body of work demonstrating that infants’ reliance on shared shape similarity to guide their inductive inferences is diminished when objects are labeled with either the same or distinct count nouns (e.g., Welder and Graham, 2001; Graham et al., 2004; Graham and Kilbreath, 2007; Keates and Graham, 2008). The results of the current study extend this literature suggesting that around 14-months, infants are beginning to establish distinct categories and use those categories to guide their inferences, even in the presence of highly perceptually similar objects.

Second, our results demonstrate that age, as an index of cognitive maturation, is an important predictor of infants’ use of distinct labels to license their inductive inferences. Thus, as infants develop, so does their ability to privilege distinct labels, even when objects are highly similar in appearance. Yet, the nature of the specific developing abilities that underlie infants’ use of distinct labels remains unclear. That is, although the ability to use distinct labels was significantly related to age, relations with measures of executive function and vocabulary were less conclusive. What might account for the lack of significant relations between measures of executive function and the use of distinct labels? We discuss different possibilities below.

In this study, infants’ performance on the working memory task was correlated only with age. As expected, working memory was unrelated to performance on the inductive task in the No Label group. As the target object remained in the infants’ line of view, infants were not required to remember any information about the object. We did predict, however, that infants who demonstrated a higher level of working memory capacity would also perform better on the inductive inference task in the Distinct Label group. This prediction was not confirmed, which may suggest that working memory does not play a role in infants’ ability to use distinct labels in an inductive inference task. Alternatively, it could be the case that the working memory task and the inductive inference task differ slightly in the type of working memory required. Based on research with adults and school-aged children, it has been argued that working memory is comprised of phonological and visual working memory (Baddeley and Hitch, 1974). In our inductive inference task infants were required to remember that two objects have different names, placing demands on both visual and phonological working memory (i.e., infants were required to associate a verbal label with a visual object). The memory task used in the current study primarily assessed infants’ visual working memory, in that infants were tasked with remembering the location of the hidden objects and retrieving it accordingly. Thus, it is possible that the use of a different memory task may have yielded significant relations with the inductive inference task. Assessing this possibility is challenging as there are, to date, no reliable measures for testing auditory and visual working memory separately with infants younger than 2-years-old (Stokes and Klee, 2009).

With respect to inhibitory control, there was no observed relation between performance on the detour-reaching task and age or vocabulary size. Further, we had predicted that infants who performed better on the inhibitory control task would rely more on the distinct label to guide their inductive inferences. Again, however, this prediction was not supported as there was no observed relation between performance on the inhibitory control task and infants’ performance of actions on the high-similarity object in the Distinct Label group. What might account for this lack of relation? To our knowledge, this is the first time that the detour-reaching task has been used to measure inhibitory control with infants aged 14- to 16-months. It is possible that the detour-reaching task was not an appropriate measure for assessing inhibition with this age group. Given, however, that there were no ceiling or floor effects on the detour-reaching task in the current study, it is unlikely that the task was either too difficult or too easy for infants. A close comparison of infants’ performance on the detour-reaching task in the current study and that of Yott and Poulin-Dubois (2012) revealed remarkably similar means and standard deviations. Taken together, this suggests that the detour-reaching task was an appropriate measure of inhibition in 14- to 16-month-olds.

Interestingly, we did find a significant, positive correlation between inhibitory control and performance on the induction task in the No Label condition. Specifically, infants who performed better on the inhibitory control measure performed significantly more target actions on the high-similarity object in the No Label group. This finding is consistent with the embodied account of early executive function development, which suggests that prospective motor control and executive functions are intertwined early in life (Gottwald et al., 2016). Specifically, the embodied account of executive function holds that low-level movement planning, such as performing an intended action on an object, is related to higher-order executive control early in life. Although we expected that infants who performed better on the inhibitory control measure to rely more on the distinct label than shared shape, we found that performance on the inhibitory control measure was related to the number of target actions performed on the high-similarity object in the No Label group. Thus, we found that performance on a complex inhibition task was related to infants’ ability to planfully execute a specific action on an object in 14- to 16-month-old infants. Perhaps complex inhibition is in the very early stages of development during these months and while this emerging ability is related to lower-level motor control, it may not yet be developed enough to underlie more sophisticated abilities, such as prioritizing distinct labels over more other cues, such as shape.

As expected, parent-reported receptive and expressive vocabulary was significantly related to age. Vocabulary size was not related to performance on the inductive inference, working memory or inhibitory control tasks. We had expected that infants with more linguistic experience would demonstrate a more sophisticated ability to use distinct labels to guide their inductive inferences. However, this lack of relation is consistent with previous research suggesting that name-based categorization is related to vocabulary size at 20-months, but not 16-months (Nazzi and Gopnik, 2000). Perhaps vocabulary size is unrelated to performance on the induction task at this stage in development, but as infants mature and their vocabulary increases, this individual difference becomes more important in detecting variability in infants’ inductive reasoning abilities.

In summary, the results of the present study demonstrate that between 14- and 16-months of age, infants begin to use distinct labels to highlight differences and carve out distinct categories, even in the presence of highly perceptually similar objects. Furthermore, infants’ use of distinct labels was related to age, but not to measures of their inhibitory control, memory or vocabulary size.

## Author Contributions

JS conducted this research in partial fulfillment on the requirements for the M.Sc. degree, under the supervision of SG. Some of the data from this experiment were included in JS’s M.Sc. thesis, submitted to the University of Calgary. Both authors contributed to the conceptualization, design, and analysis of the data and to the preparation of the manuscript.

## Conflict of Interest Statement

The authors declare that the research was conducted in the absence of any commercial or financial relationships that could be construed as a potential conflict of interest.
